# Influence of Aqueous Phase Composition on Double Emulsion Stability and Colour Retention of Encapsulated Anthocyanins

**DOI:** 10.3390/foods11010034

**Published:** 2021-12-23

**Authors:** Damien A. Sebben, Stephanie V. MacWilliams, Long Yu, Patrick T. Spicer, Vincent Bulone, Marta Krasowska, David A. Beattie

**Affiliations:** 1 Future Industries Institute, UniSA STEM, Mawson Lakes Campus, University of South Australia, Mawson Lakes, SA 5095, Australia; damien.sebben@gmail.com (D.A.S.); Stephanie.MacWilliams@unisa.edu.au (S.V.M.); 2 School of Agriculture, Food and Wine, The University of Adelaide, Adelaide, SA 5064, Australia; Long.Yu@adelaide.edu.au (L.Y.); Vincent.Bulone@adelaide.edu.au (V.B.); 3 Complex Fluids Group, School of Chemical Engineering, UNSW, Sydney, NSW 2052, Australia; p.spicer@unsw.edu.au; 4 Department of Chemistry, Division of Glycoscience, KTH Royal Institute of Technology, 114 28 Stockholm, Sweden

**Keywords:** anthocyanins, electrolyte composition, encapsulation, double emulsion stability, gelation, pectin

## Abstract

Water-in-oil-in-water (W_1_/O/W_2_) emulsions (double emulsions) have often been used for the encapsulation of bioactive compounds such as anthocyanins. Instability of both anthocyanins and double emulsions creates a need for a tailored composition of the aqueous phase. In this work, double emulsions with a gelled internal water phase were produced and monitored over a 20-day storage period. The effect of the electrolyte phase composition (varying electrolyte components, including adipic acid, citric acid, and varying concentration of potassium chloride (KCl)) on anthocyanin and double emulsion stability was analysed using colour analysis, droplet sizing, and emulsion rheology. The effect of electrolytes on colour retention was shown to differ between the primary W_1_/O emulsion and the secondary W_1_/O/W_2_ emulsion. Furthermore, droplet size analysis and emulsion rheology highlighted significant differences in the stability and structural behaviour of the emulsions as a function of electrolyte composition. In terms of colour retention and emulsion stability, a citrate-buffered system performed best. The results of this study highlight the importance of strict control of aqueous phase constituents to prevent anthocyanin degradation and maximise double emulsion stability. Additional experiments analysed the effect of pectin chemistry on the anthocyanin colour retention and leakage, finding no conclusive difference between the unmodified and amidated pectin.

## 1. Introduction

Anthocyanins are a class of naturally occurring polyphenolic compounds responsible for the vibrant blue, red, and purple colours of many fruits and vegetables, which have also been used as a natural colourant in the food industry (where it is given the food colourant code E163 [1], and is authorised for use in the European Union, Australia, and New Zealand, and can be found in common supermarket products such as baked goods, chocolates, and energy drinks). Anthocyanins are also reported to have a number of health benefits including anti-cancer, anti-inflammatory, and anti-oxidant properties [2,3,4]. However, the colour and bioactivity of anthocyanins when deployed as food colourants are often diminished due to degradation and/or oxidation resulting from chemical and physical factors such as temperature, pH, and exposure to UV light, metal ions, and ascorbic acid [5,6,7,8]. As such, encapsulation methods are routinely employed to protect anthocyanins and other pigments [9] to preserve their beneficial properties during food production and storage.

One potential vehicle for anthocyanin encapsulation is the double emulsion. Water-in-oil-in-water (W_1_/O/W_2_) double emulsions are produced through the dispersion of a primary emulsion (W_1_/O) in a secondary aqueous phase (W_2_) to produce the secondary emulsion. The composition and properties of the two aqueous phases can be altered to allow for better stabilisation and protection of the contents encapsulated within the internal water droplets. Microencapsulation of bioactive molecules by means of a double emulsion has previously been shown to protect specific molecules (e.g., vitamin B_12_, resveratrol, curcumin) from degradation and/or interference from other components [10]. Several studies have also shown that inclusion of anthocyanin molecules in the internal aqueous phase of a double emulsion can protect them from changes in pH [11], temperature [12], and during simulated mouth and gastric digestion [13,14,15,16].

Despite their protective ability, double emulsions are very susceptible to breakdown due to thermodynamic instability. The presence of two oil-water interfaces requires both lipophilic emulsifiers (those that are added to the oil phase and adsorb to the interface from the oil) and hydrophilic emulsifiers (those that are added to a water phase and adsorb to the interface from the water) to stabilise the primary and secondary emulsions, respectively. Osmotic pressure gradients can also exist between the internal and external aqueous phase, resulting in swelling (and eventual rupture) or coalescence of the internal emulsion droplets, leading to degradation of the encapsulated ingredient when it is expelled into the external aqueous phase [10,12,17]. In food systems, challenges also arise from the required use of food safe emulsifiers.

Gelation of the internal aqueous phase has been effective in counteracting the instability mechanisms resulting from osmotic pressure gradients and has also been shown to prevent internal droplet coalescence [10,18]. Pectin is often used as a gelling agent for the internal water droplets and has been reported to form molecular complexes with anthocyanins through hydrogen bonding or hydrophobic interactions, which has resulted in improved anthocyanin colour stability [8,19,20,21,22]. The addition of electrolytes such as acids and salts controls the osmotic pressure gradient between the internal and external aqueous phases of a double emulsion [10]; however, the composition of the electrolytes can also influence the colour stability of anthocyanins. Hubbermann et al. reported that acetic and adipic acids had the least detrimental effects on anthocyanin colour stability, while salt concentrations above 3% (≈0.5 M) resulted in loss of anthocyanin colour over longer storage periods [21]. It is evident that a careful balance of electrolytes and stabilisers is required to produce a double emulsion with long-term physical stability while not compromising the preservation of anthocyanins colour and biological properties.

Colour retention within an anthocyanin encapsulation system has been used as a measure of its ability to protect anthocyanins from physical or chemical degradation [8,23,24]. The colour expressed by anthocyanins is dependent on the structural forms present in solution. The red-coloured flavylium ion dominates the equilibrium at low pH values. Increasing pH results in competitive reactions forming either the unstable, blue-coloured quinoidal base or the more stable hemiketal. The hemiketal structure is in equilibrium with the yellow-coloured *cis*-chalcone, which in turn isomerises to form the *trans*-chalcone [20]. The CIE L*a*b* colour scale can be used to analyse colour changes resulting from anthocyanin degradation. The a* (red-greenness) and b* (blue-yellowness) parameters are useful in determining the stability of anthocyanins based on the colour of the system. In particular, the a* parameter is an indicator of anthocyanin colour stability [21], however, both the a* and b* parameters should be considered concurrently to explain the mechanisms for colour change. The a* and b* parameters have been shown to exhibit a linear correlation with the degradation of anthocyanins [7].

In this study, anthocyanins from bilberry and blackcurrant extracts were encapsulated in the internal aqueous phase of a W_1_/O/W_2_ double emulsion. The physical stability of the double emulsions and their ability to encapsulate and protect the anthocyanins against degradation when prepared with different electrolyte compositions was investigated through colour analysis, particle size measurements, encapsulation efficiency, and rheological studies over 20 days. To the best of our knowledge, this is the first study to investigate the effect of electrolyte composition and pectin chemistry on both the physical stability of a double emulsion and the colour stability of the encapsulated anthocyanins.

## 2. Materials and Methods

### 2.1. Chemicals

Commercially available berry anthocyanin extract, MEDOX^®^, was purchased from Medpalett AS (Sandnes, Norway). Cyanidin chloride (≥96%) and cyanidin-3-O-glucoside chloride (≥97%) were purchased from Extrasynthese (Genay, France). Olive oil was purchased from a local supermarket (Adelaide, Australia). Unmodified (CU-L 021/20) and amidated (CU-L 022/20) pectin containing no additives were supplied in-kind by Herbstreith & Fox GmbH & Co. KG (Neuenbürg, Germany) and polyglycerol polyricinoleate (PGPR), GRINDSTED^®^ PGPR 90 were supplied in-kind by Danisco (Pulau Pinang, Malaysia). Soy lecithin (>97% phospholipids) and methanol (HPLC analytical grade) was purchased from ThermoFisher Scientific (Scoresby, Australia). Sodium carboxymethyl cellulose (CMCNa, mw = 250,000), potassium sorbate (≥99%), adipic acid (99%), and trifluoroacetic acid (TFA, HPLC analytical grade) were purchased from Sigma Aldrich (Castle Hill, Australia). Citric acid monohydrate, trisodium citrate dihydrate, potassium chloride, and calcium chloride dihydrate were purchased from Chem Supply (Gillman, Australia). Volumetric grade KOH and HCl solutions for pH adjustment were purchased from Merck (Darmstadt, Germany). Solutions were prepared using Milli-Q water (Millipore, MA, USA); resistivity 18.2 MΩ.cm; surface tension 72.4 mN.m^−1^ at 22.0 °C; total organic carbon below 4 ppb.

### 2.2. Methods

#### 2.2.1. Preparation of Water-in-Oil-in-Water (W_1_/O/W_2_) Emulsions

Anthocyanin-loaded W_1_/O/W_2_ emulsions were prepared using a two-stage emulsification process [15,22]. An initial anthocyanin-loaded water-in-oil (W_1_/O) emulsion was prepared, followed by the addition of a second water phase (W_2_) and subsequent emulsification to produce the W_1_/O/W_2_ emulsion. Four different background electrolyte solutions were used in this work: 0.1 M citrate buffer, 0.1167 M adipic acid, 0.35 M KCl, and 0.0075 M KCl (note: adipic acid was solubilised using a solution at slightly elevated pH, using the addition of potassium hydroxide, and then the pH was reduced to the value used in emulsion preparation). All electrolyte solutions were adjusted to pH 3.5 and contained 0.2 wt% potassium sorbate as an antimicrobial agent (even though the pH of all electrolytes was initially adjusted to 3.5, for KCl solutions the final pH values were higher once the final W_2_ solutions were prepared (see Table S1)). The nature of the weak electrolytes means that the solution conductivity was used for comparison instead of ionic strength. The oil phase was prepared by adding 2.5 wt% PGPR to olive oil followed by stirring (using a stirring hotplate and magnetic stirrer bar) at 60 °C for at least 30 min to ensure complete dissolution. The W_1_ phases were prepared by combining two separate solutions: a pectin solution and an anthocyanin solution. The 3.0 wt% pectin solutions were prepared by dissolving either amidated or unmodified pectin in the background electrolyte via stirring at 95 °C. Solutions of anthocyanin (40 mg/mL) were prepared by dissolving the extract in background electrolyte containing Ca^2+^ ions at a concentration equal to 0.25 wt% (for amidated pectin) or 0.50 wt% (for unmodified pectin) of the total pectin mass (once the pectin and anthocyanin solutions were combined). These Ca^2+^ concentrations yield a solution that flows at room temperature and gels upon cooling. Once pectin hydration/dissolution was complete, the solutions were cooled for 2 min before addition to the anthocyanin solution at a 1:1 weight ratio. W_2_ phases were prepared by adding 1.5 wt% CMCNa and 1.5 wt% soy lecithin in background electrolyte and stirring at 60 °C until completely hydrated/dissolved.

Primary W_1_/O (water fraction, φ_W1_, of 30%) emulsions were prepared by adding 9 g of the combined W_1_ phase to 21 g oil phase prior to emulsification at 20,000 rpm for 3 min using a Unidrive X 100 D rotor stator homogeniser (Ingenieurbüro CAT, Ballrechten-Dottingen, Germany). Emulsions were stirred briefly with a clean glass pipette before being homogenised for a further 1 min at 20,000 rpm. The prepared W_1_/O emulsions were placed in a fridge (T = 4 °C) for 1.5 h to ensure pectin gelation.

Secondary W_1_/O/W_2_ emulsions (primary emulsion fraction, φ_W1/O_, of 30%) were prepared by adding 18 g of the W_1_/O emulsion (with a gelled internal W_1_ phase) to 42 g of the W_2_ phase. The two phases were then homogenised at 16,000 rpm for 3 min. Emulsions were placed into clean, amber glass vials, sealed with screw-top caps, and stored in the dark at 25.0 ± 0.1 °C in an incubator (Memmert GmbH + Co. KG, Büchenbach, Germany).

Each emulsion system was prepared in triplicate as independent repeats. The three independent emulsions were used in all subsequent experiments.

#### 2.2.2. Emulsion Microstructure

Confocal laser scanning micrographs of a W_1_/O/W_2_ emulsion (amidated pectin, 0.1 M citrate buffer system) diluted (1:1 *w*/*w*) in 0.1 M citrate buffer and stained with a lipid soluble fluorescent dye, Nile red (Sigma Aldrich, Castle Hill, Australia), were taken on days 1, 2, and 5. Ten µL of 1 mg/mL Nile red in ethanol solution was added to 300 µL of diluted emulsion and vortexed briefly (≈10 s). Approximately 2 µL of the diluted, stained emulsion was sandwiched between two clean, microscope cover slips (Gerhard Menzel GmbH, Brunswick, Germany). Images were taken using a Zeiss LSM 710 Elyra PS.1 microscope fitted with a water immersion objective, C-Apochromat 63×/1.2 W Korr M27 (ZEISS Group, Oberkochen, Germany). The excitation wavelength was 514 nm and the detection wavelength ranged from 539 to 753 nm.

#### 2.2.3. Droplet Size Analysis

Emulsion droplet size distributions were determined using a Malvern Mastersizer 3000 equipped with a Hydro-LV sampling attachment (Malvern Panalytical, Malvern, UK). The Hydro-LV attachment was rinsed five times with Milli-Q water prior to and between samples, before being emptied and filled with the background electrolyte relevant to the emulsions being studied. The stirrer speed was kept constant at 1000 rpm and no sonication was used. The refractive indices of the oil and aqueous phases were set to 1.45 and 1.33, respectively, while the absorbance value was held at 0.01.

#### 2.2.4. Colour Stability Measurements

Evaluation of the colour parameters over time was undertaken following the CIE L*a*b* parameters (Figure S2, Supplementary Material) using a HunterLab UltraScan VIS spectrophotometer (Hunter Associates Laboratory Inc., Reston, VA, USA). The samples were placed in 10 mm quartz cuvettes (Starna Scientific, Baulkham Hills, Australia), placed in the instrument for light exposure, and reflected light was collected (at 8° to the incident light angle). The a* (red-greenness), and b* (blue-yellowness) parameters were used to calculate chroma (c*) and hue angle (*h°*) via Equations (1) and (2), respectively:

(1) $$c^{*}={\left(a^{*2}+b^{*2}\right)}^{1/2}$$

(2) $$h^{{}^{\circ}}=tan^{-1}\left(b^{*}/a^{*}\right)$$

#### 2.2.5. Emulsion Rheology

The rheological behaviour of the emulsions as a function of time and electrolyte composition was monitored using a Kinexus pro+ rotational rheometer (Malvern Panalytical, Malvern, UK). A cone and plate geometry (cone diameter of 40 mm, angle of 4°, and a gap of 0.1584 mm) was used and all measurements were conducted at 25.0 °C. Strain sweeps were conducted in the range 0.1–100% with the frequency held constant at 1 Hz. Shear rate ramps were conducted from 1–100 s^−1^ over a 5 min time period.

#### 2.2.6. Anthocyanin Encapsulation Efficiency and Leakage

The encapsulation efficiency (EE) of W_1_/O/W_2_ emulsions containing either unmodified or amidated pectin as well as the leakage of anthocyanins from the W_1_ phase to the W_2_ phase was determined. Prepared emulsions were diluted 50% in the background electrolyte then centrifuged at 20,000× *g* for 15 min. The aqueous phase was carefully removed and filtered through a 0.80 µm cellulose acetate syringe filter (Sartorius, Göttingen, Germany). The aqueous samples were then placed onto 0.22 µm cellulose acetate centrifuge filters (Corning Incorporated, Corning, NY, USA) and centrifuged at 15,500× *g* for 45 min to remove suspended solids. The clear anthocyanin solutions were then analysed using a ThermEvo UV–Vis spectrophotometer (Thermo Fisher Scientific, Waltham, MA, USA) at a wavelength of 520 nm. The anthocyanin concentration was determined from a calibration curve of known concentrations of MEDOX^®^ (London, UK) (concentration of anthocyanin in the stock material was determined by HPLC, and this analysis is given in the Supplementary Materials). The *EE* of each emulsion, defined as the percentage of anthocyanins retained within the W_1_ phase following emulsion production, was calculated as follows:

(3) $$EE\left(\%\right)\;=\;\left(TAC-FAC\right)/\left(TAC\right)\times 100$$

where *TAC* is the total anthocyanin content added to the W1 phase and *FAC* is the free anthocyanin content in the W_2_ phase (with 1:1 *w*/*w* dilution considered).

## 3. Results and Discussion

### 3.1. Emulsion Microstructure

**Figure 1:**
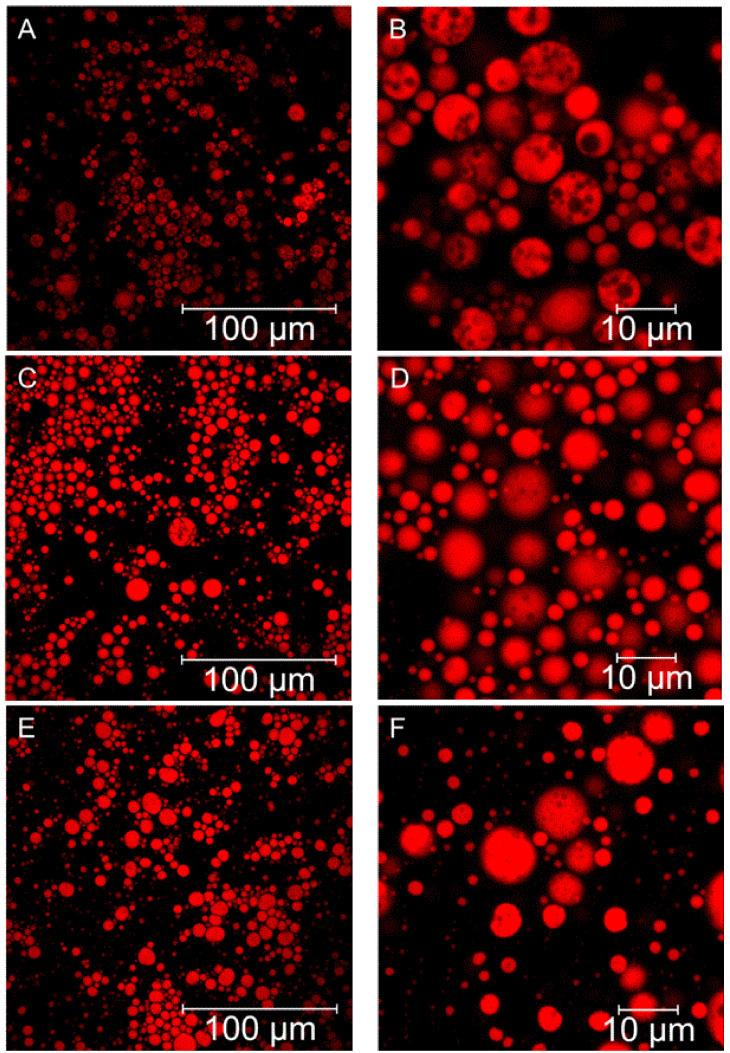
Confocal laser scanning micrographs of a W_1_/O/W_2_ emulsion with an amidated pectin-gelled W_1_ phase, 0.1 M citrate buffer as the background electrolyte, and an oil phase dyed with Nile red. Images of the emulsion on day 1 (Panel (**A**,**B**)), day 2 (Panels (**C**,**D**)), and day 5 (Panels (**E**,**F**)) are shown. Panels (**B**,**D**,**F**) are magnified images from the same sample as that of Panels (**A**,**C**,**E**), respectively.

Confocal laser scanning microscopy (CLSM) (Carl Zeiss AG, Oberkochen, Germany) was used to study the structure of the double emulsions [10,25,26,27]. Figure 1A,B are images of a W_1_/O/W_2_ emulsion, formulated with 0.1 M citrate buffer and amidated pectin in the W_1_ phase, on day 1 (Figure 1B shows a magnified view of the same sample). The oil droplets (red regions) contained a significant amount of aqueous phase, W_1_, as evidenced by the darker unstained spherical regions of varying sizes within the oil droplets. The oil droplets seemed to form ‘rafts’ of aggregates, consistent with previous studies using soy lecithin and CMCNa [15]. Figure 1C,D show the same emulsion on day 2, where a reduction in the amount of encapsulated W_1_ droplets was apparent, suggesting that W_1_ droplets are being expelled from the oil phase. The same emulsion on day 5 (Figure 1E,F) did not appear to differ markedly from the emulsion on day 2; loss of encapsulated W_1_ droplets occurred primarily within the first 24 h. In addition, the W_1_ droplets contained within the oil phase appear to be smaller on days 2 and 5 than on day 1. The higher conductivity of the W_1_ phase compared to the W_2_ phase (Table S1, Supplementary Materials) means that there is likely a movement of water from W_2_ to W_1_, causing the W_1_ droplets to swell. Once these W_1_ droplets reach a critical volume, they are they likely to be expelled from the oil droplets. Figure 1E,F (day 5) show differences in the oil droplet shape compared with days 1 and 2. The droplets appeared irregularly shaped on day 5, instead of the spherical shape seen on days 1 and 2.

### 3.2. Droplet Size Analysis

**Figure 2:**
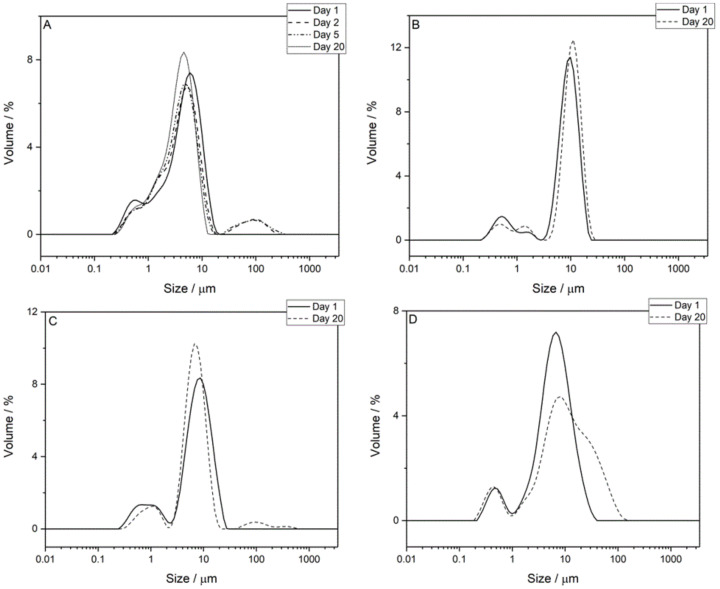
Representative droplet size distributions for: (**A**) 0.1 M citrate buffer; (**B**) 0.1167 M adipic acid; (**C**) 0.35 M KCl; and (**D**) 0.0075 M KCl. Data for days 1, 2, 5, and 20 are shown for the 0.1 M citrate buffer emulsion, and data for days 1 and 20 are shown for the 0.1167 M adipic acid, 0.35 M KCl, and 0.0075 M KCl emulsions.

Figure 2 shows the droplet size distributions from a representative sample for each of the four W_1_/O/W_2_ emulsion systems. Days 1, 2, 5, and 20 are shown for the 0.1 M citrate emulsion (Figure 2A) while days 1 and 20 are shown for the 0.1167 M adipic acid, 0.35 M KCl, and 0.0075 M KCl emulsions (Figure 2B–D). The chemistry and concentration of the background electrolyte have an impact on the droplet size at the time of formulation and during storage. CLSM data from Figure 1 suggest there was significant crossover of oil and water droplets within the size distribution dataset for the 0.1 M citrate buffer emulsions; there were gelled W_1_ droplets up to 5 µm in diameter as well as small oil droplets 1 µm in diameter. As such, the two peaks are not strictly representative of separate droplet phases. However, it is likely that the lower and upper regions of the size distributions result primarily from W_1_ and oil droplets, respectively, considering the emulsification conditions.

Similar droplet size behaviour was noted for the 0.1 M citrate buffer and 0.35 M KCl systems in Figure 2A,C, respectively, with a small decrease in the upper size regions for both the 0.1 M citrate buffer (5.92 to 4.58 µm) and 0.35 M KCl (8.68 to 6.72 µm), albeit the size of oil droplets in 0.35 M KCl was larger by over 45%. Additionally, there were broad peaks between ~30 and 500 µm for the 0.1 M citrate buffer (on days 2 and 5) and 0.35 M KCl emulsion (day 20). These peaks were inconsistent and irregular and are likely due to the presence of bubbles and/or large aggregates of droplets. The similarities between the 0.1 M citrate buffer and 0.35 M KCl was confirmed by the volume-weighted diameter percentiles, d (0.1), d (0.5), and d (0.9), plotted as a function of storage time in Figure S1A,C (Supplementary Materials), where the d (0.5) and d (0.9) decreased over time. For the 0.35 M KCl system, there was no discernible change in the d (0.1). However, the 0.1 M citrate buffer system showed an increase from 0.88 ± 0.05 µm to 1.02 ± 0.06 µm. Previous studies of double emulsions have also noted a decrease in oil droplet size over time [12,18,28]. The mechanism was reported as either the transport of water molecules from the W_1_ droplets to W_2_ phase, driven by an osmotic pressure gradient [26], or complete expulsion of W_1_ droplets into the W_2_ phase [28]. In the case of the 0.1 M citrate buffer and 0.35 M KCl emulsion systems, the conductivity within the W_1_ phase was higher than that of the W_2_ phase (Table S1), suggesting that the movement of water would likely be from W_2_ to W_1_, thus increasing the W_1_ droplet size. An increase in the W_1_ droplet size resulting from water transport and/or droplet aggregation may explain the upward shift in the lower peaks for both the 0.1 M citrate buffer and 0.35 M KCl emulsions (Figure 2A,C). The size distributions on days 1, 2, and 5 for the 0.1 M citrate emulsion were in keeping with the CLSM data in Figure 1, where the greatest change occurred in the first 24 h of storage, followed by minimal change between days 2 and 5. The smaller droplets showed an increase in size, while the larger (predominantly oil) droplets showed a decrease. These changes in size distribution support a theory of osmotic-driven swelling of the W_1_ droplets, followed by subsequent expulsion into the W_2_ phase. Coalescence of the smaller oil droplets could also account for the change in size distribution for the lower size region, however, coalescence of the larger oil droplets and subsequent creaming/destabilisation of the emulsions was not seen, indicating good stability of the oil phase.

An overall increase was noted for the upper size regions of the 0.1167 M adipic acid and 0.0075 M KCl systems. The size distribution for the 0.1167 M adipic acid system showed a 13.71% increase in upper peak position over the 20-day storage period, from 9.85 to 11.20 µm, while the smaller droplets within the system also seemed to be increase in diameter; the lower peak (0.52 µm) of the distribution decreased in volume while the higher peak (1.45 µm) increased in volume. However, the volume-weighted diameter percentile data for the three 0.1167 M adipic acid emulsions (Figure S1B) did not provide evidence of a significant increase in d (0.5) or d (0.9). There was a notable increase in d (0.1) from 1.01 ± 0.05 µm to 1.38 ± 0.16 µm. The mechanism for this volume increase for lower peaks is likely due to W_1_ droplet aggregation and/or small oil droplet coalescence. Swelling of W_1_ is less likely as the conductivity of W_1_ was less than that of W_2_. The absence of change in the d (0.5) and d (0.9) values suggests that, unlike the 0.1 M citrate buffer and 0.35 M KCl emulsions, there was either minimal expulsion of gelled W_1_ droplets into the W_2_ phase or there an overall increase in the size of the larger (predominantly oil) droplets due to coalescence, which offset any decrease that would have otherwise been noticed due to W_1_ droplet expulsion. A significant shift in the upper peak was noted for the 0.0075 M KCl system in Figure 2D and confirmed by the volume-weighted diameter percentile data in Figure S1D. The initially narrow distribution for 0.0075 M KCl centred around 6.72 µm shifted upward to 7.63 µm, and an additional shoulder around 27.37 µm appeared (Figure 2D). The d (0.5) increased from 7.98 ± 1.37 µm to 12.10 ± 1.96 µm and the d (0.9) increased significantly from 20.10 ± 3.22 to 46.00 ± 4.83 µm. The lower peak around 0.46 µm remained relatively consistent. The 0.0075 M KCl system showed significant coalescence between oil droplets during the storage period, but there was little evidence to suggest that any appreciable aggregation or swelling of the W_1_ phase was occurring.

The droplet size analysis depicts differences in droplet behaviour as a function of electrolyte composition. For the 0.1 M citrate buffer, 0.1167 M adipic acid, and 0.35 M KCl systems, there was an increase in the size of the smaller droplets (lower size region). The likely explanation is an osmotic pressure-driven transfer of W_2_ to W_1_, causing swelling of the internal water droplets. There is also a possibility that coalescence of small oil droplets (seen in Figure 1) causes the increase in the lower peaks for these two systems. The 0.1 M citrate buffer and 0.35 M KCl systems showed a decrease in the size of the larger droplets (upper size region). Expulsion of W_1_ droplets caused a decrease in oil droplet size. For the 0.1167 M adipic acid emulsions, no change in the upper peak was noted, suggesting that coalescence between oil droplets may offset any observed decrease in oil droplet size resulting from W_1_ droplet expulsion. The 0.0075 M KCl system showed a significant change in oil droplet size distribution that led to eventual destabilisation (creaming) of the W_1_/O/W_2_ emulsions, indicating that the lower concentration of electrolyte does not support W_1_/O/W_2_ emulsion stability. The nature of the electrolyte also has some bearing on the droplet size evolution following production. In terms of the W_1_ phase, this is likely due to the interactions of the electrolyte with pectin. Slight changes in the extent of gelation may influence how the W_1_ droplets are formed during the emulsification procedure. According to Frank et al., the addition of a hydrocolloid to the inner water phase affects droplet formation during homogenisation [22]. Thus, any changes to W_1_ viscosity due to electrolyte composition may be the cause of the difference in initial W_1_ droplet size distribution.

### 3.3. Colour Analysis

**Figure 3:**
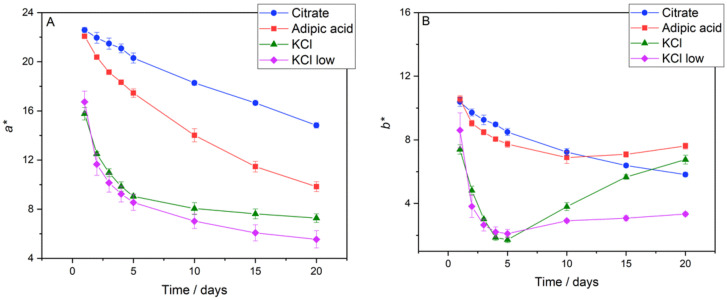
The CIE L*a*b* colour parameters a* (Panel (**A**)) and b* (Panel (**B**)) for W_1_/O/W_2_ emulsions. The 0.1 M citrate buffer system is represented by circles, 0.1167 M adipic acid by squares, 0.35 M KCl by triangles, and 0.0075 M KCl by diamonds.

The CIE L*a*b* colour parameters were measured for each W_1_/O/W_2_ and W_1_/O sample over the 20-day storage period to monitor colour changes for all studied systems. Figure 3 shows the a* (Panel A) and b* (Panel B) parameters for the W_1_/O/W_2_ emulsions formulated with different electrolytes as a function of storage time. It is immediately apparent that the a* parameter decreased markedly over the 20-day storage period, consistent with other studies [12,23]. In contrast, colour retention was better in the W_1_/O emulsion and W_1_/O/W_2_ emulsions for all electrolyte systems (see Figure S3A). In terms of the W_1_/O/W_2_ emulsions, the 0.1 M citrate buffer and 0.1167 M adipic acid systems showed the highest values of a* at the time of formulation (22.58 ± 0.21 and 22.08 ± 0.10, respectively). Both systems showed a decrease in a*, albeit the decrease over the 20-day period was significantly smaller for the 0.1 M citrate buffer emulsions, dropping to 14.82 in a near linear trend. The decrease in a* for the W_1_/O/W_2_ emulsion formulated with 0.1167 M adipic acid was more significant, dropping down to 7.61 on day 20; a 21.10% greater decrease in a* than the citrate buffer emulsions. Both W_1_/O/W_2_ emulsions formulated with KCl showed a much faster (and exponential) decrease in a* with 0.35 M KCl, providing a marginally better colour retention from day 5 onwards than 0.0075 M KCl. Overall, the decrease in a* for the 0.35 M KCl and 0.0075 M KCl emulsion systems was 19.50% and 32.49% greater, respectively, compared to the best performing 0.1 M citrate buffer formulation. Interestingly, the 0.1167 M adipic acid W_1_/O emulsions showed greater colour retention (in terms of a*) than the 0.1 M citrate buffer emulsions (17.13 and 18.38% decrease, respectively). Additionally, the decrease in a* for the 0.35 M KCl and 0.0075 M KCl W_1_/O/W_2_ emulsions was 11.77 % and 45.81 % greater than the respective W_1_/O emulsions.

The b* values of the W_1_/O/W_2_ emulsions reflect a different behaviour to that of the a* values (see Figure 3B). The 0.1 M citrate buffer and 0.1167 M adipic acid systems were once again similar when the emulsions were first formulated, giving b* values of 10.38 ± 0.27 and 10.56 ± 0.22, respectively. In this instance, the 0.1 M citrate buffer emulsions underwent a gradual decline in b* intensity, whereas both KCl systems and 0.1167 M adipic acid emulsions showed a period of decline over five and 10 days, respectively, before their b* values began to increase. Anthocyanins are known to degrade to produce yellowish-brown coloured compounds [12,21,29], so the increasing amount of degradation products is likely to contribute to the increase in b* intensity. An increase in b* intensity relative to a* creates an overall shift in colour towards yellow/brown hues (Equation (2)). Similar to the a* values, the b* values for the 0.1 M citrate buffer and 0.1167 M adipic acid W_1_/O emulsions decreased less over 20-days than their W_1_/O/W_2_ counterparts (Figure S3B). The b* value decreased significantly (65.14 %) for the 0.35 M KCl W_1_/O emulsions compared to only 8.62% for the W_1_/O/W_2_ emulsions, while the opposite trend occurred for the 0.0075 M KCl emulsions; b* decreased by 19.03% for the W_1_/O emulsions and 61.24% for the W_1_/O/W_2_ emulsions.

**Figure 4:**
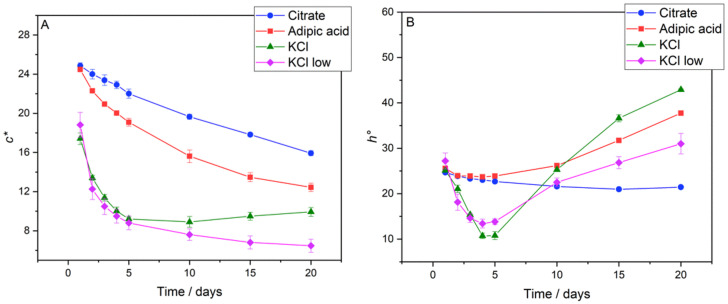
Chroma (c*, Panel (**A**)) and hue angle (*h°,* Panel (**B**)) for W_1_/O/W_2_ emulsions, calculated from the data in Figure 3. The 0.1 M citrate buffer system is represented by circles, 0.1167 M adipic acid by squares, 0.35 M KCl by triangles, and 0.0075 M KCl by diamonds.

To better understand the overall effect of concurrent changes in a* and b*, the colour parameters chroma (c*, also termed colour intensity) and hue angle (*h°*) were calculated from Equations (1) and (2), respectively. The c* vs. storage time plots for the W_1_/O/W_2_ emulsions in Figure 4A showed a decrease in vibrance for all systems over the 20-day period. The c* analysis for the W_1_/O emulsions is presented in Figure S4A. Overall, colour intensity (c*) diminishing is a likely result of anthocyanin concentration depletion. The c* values for 0.1 M citrate buffer and 0.1167 M adipic acid solution were similar at the time of production, being 24.86 ± 0.31 and 24.48 ± 0.19, respectively, because of their similar a* and b* values (Figure 3A,B). Both KCl systems had lower c* values at the time of production; 0.35 M KCl had a c* value of 17.41 ± 0.58 and 0.0075 M KCl was 18.82 ± 1.28. The 0.35 M KCl sample showed a decrease in c* until day 10 to 8.91 ± 0.55, before c* began to slightly increase, reaching 9.93 ± 0.45 at day 20. This increase in c* after day 10 for the 0.35 M KCl W_1_/O/W_2_ emulsions corresponds to the increase in b* seen in Figure 3B. The three other emulsions experienced a decrease over the 20-day period, with 0.1 M citrate buffer experiencing the smallest decrease (35.93%), followed by 0.1167 M adipic acid (49.19%) and 0.0075 M KCl (65.60%). For the W_1_/O emulsions, c* decreased over time for all electrolyte systems, with the 0.1167 M adipic acid and 0.1 M citrate buffered emulsions showing better retention, both decreasing by 16.18%. With the exception of the 0.35 M KCl system, the drop in c* was notably less for the W_1_/O emulsions than the W_1_/O/W_2_ emulsions.

The *h°* vs. storage time plots (Figure 4B) for the W_1_/O/W_2_ emulsions showed relatively stable colour hue for the 0.1 M citrate buffer system, experiencing a decrease from 24.69 ± 0.37° to 21.43 ± 0.17° (a change of 13.21%). As expected, *h°* values for the 0.1167 M adipic acid system and both KCl systems increased over the 20-day period due to increasing b* values (Figure 3B). The increase in *h°* for the 0.1167 M adipic acid system was from 25.57 ± 0.36° to 37.74 ± 0.44° (a change of 47.57%). The effect of ionic strength on anthocyanin colour stability can be highlighted by the differences in *h°* shift between the two KCl systems. The 0.35 M KCl emulsions experienced a shift in *h°* from 25.14 ± 0.17° to 42.90 ± 0.19°, an increase of 70.66%, compared to a 13.98% shift in *h°* for the 0.0075 M KCl emulsions, from 27.21 ± 1.76° to 31.01 ± 2.25°. Increases in *h°* of 9.57%, 3.75%, and 2.09% were experienced by the 0.1 M citrate buffer, 0.1167 M adipic acid, and 0.0075 M KCl W_1_/O emulsions, respectively (Figure S4B). The *h°* values for the 0.35 M KCl W_1_/O emulsion were significantly lower, and there was an overall decrease of 37.59% during the 20-day storage period.

The results of the colour analysis highlight interesting effects of electrolyte composition and ionic strength on the colour retention of anthocyanins in both the W_1_/O and W_1_/O/W_2_ emulsions. While 0.1167 M adipic acid and 0.0075 M KCl appeared to promote colour retention in W_1_/O emulsions, equal to 0.1 M citrate buffer, the 0.1 M citrate buffer system stood out when a secondary emulsification step occurred. In a W_1_/O emulsion, the colour retention was better for all electrolytes compared with the corresponding W_1_/O/W_2_ emulsions. The difference in colour retention between these two emulsion types can be explained by the different composition of the W_1_ and W_2_ phases. W_1_ phases contained 1.5 wt% amidated pectin and ~0.001 M CaCl_2_ in addition to the background electrolyte, with pectin exhibiting a protective effect against anthocyanin degradation.

A study by Hubberman et al. investigated the effect of food acids/acidity regulators on anthocyanin degradation in solution. The results showed that adipic acid allowed for greater colour retention than citric acid (both solutions made to 0.2 M, pH 3.9) [21]. However, our data for W_1_/O/W_2_ emulsions showed the reverse trend with citrate buffer assuring the best colour retention. The pH of the W_2_ phases for the 0.1 M citrate buffer and 0.1167 M adipic acid systems were 3.8 and 4.0, respectively, at the time of production. This value may differ on day 20, but it is not expected that it could account for the changes seen in a*. The presence of 0.35 M or 0.0075 M KCl is even more detrimental to colour retention. More significant changes in W_2_ pH for the non-buffered KCl (5.1 for 0.35 M KCl and 5.8 for 0.0075 M KCl) systems would likely contribute to the differences in the colour parameters.

### 3.4. Rheology

**Figure 5:**
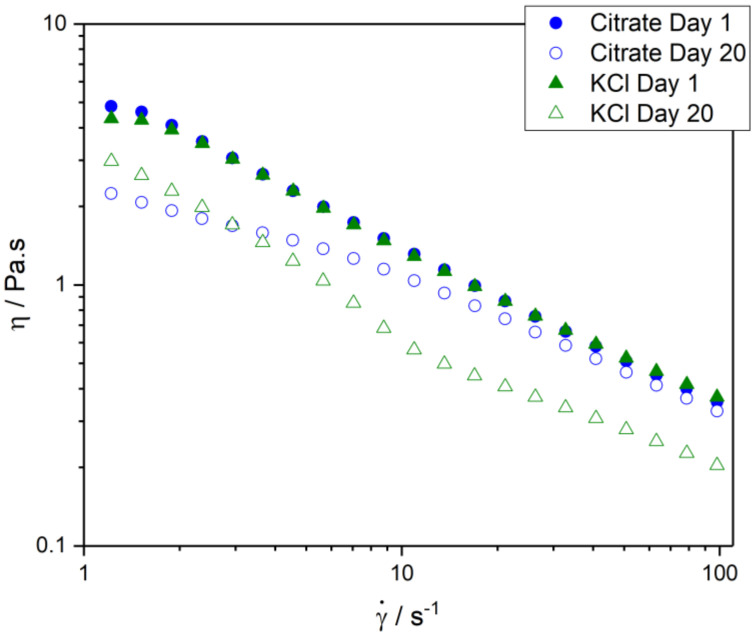
Flow curves between shear rates of 1 and 100 s^−1^ for the 0.1 M citrate buffer (blue circles) and 0.35 M KCl (green triangles) emulsions on days 1 (closed symbols) and 20 (open symbols).

Shear rate ramps (flow curves) were performed on each emulsion for days 1 to 20. All flow curves showed a decrease in viscosity as a function of shear rate, indicating that all systems had non-Newtonian shear-thinning flow behaviour, consistent with other double emulsion studies [16,28,30]. According to McClements, shear-thinning can occur due to altered spatial distribution of particles; non-spherical particle alignment with a flow field; removal of bound solvent molecules; and the deformation of flocs [25]. Flow curves from a single representative emulsion of both the 0.1 M citrate buffer and 0.35 M KCl systems are presented in Figure 5 for days 1 and 20. The results highlight the differences in rheological properties that develop during emulsion storage. At the time of measurement on day 1, the systems behaved similarly to the two datasets in close agreement. However, by the end of the storage period, the nature of the emulsions had changed depending on the electrolyte system. The 0.1 M citrate buffer, 0.35 M KCl, and 0.0075 M KCl emulsions became less viscous over time (Figure 5 and Figure S5). Interestingly, the 0.1167 M adipic acid system showed an increase in viscosity over the first 10 days of storage, followed by a gradual decrease from days 10 to 20 (Figure S5, only days 1 and 20 shown). Although the viscosity decreased from days 10 to 20, the viscosity was still higher at day 20 than day 1.

To better summarise the effect of storage time on emulsion behaviour, all experimentally obtained flow curves were fitted with the Ostwald de-Waele (power-law) model as a function of shear rate, $\dot{\gamma }$, as shown in Equation (4):

(4) $$\eta =k\cdot {\dot{\gamma }}^{n-1}$$

where *k* is the consistency index and *n* is the power law exponent.

**Figure 6:**
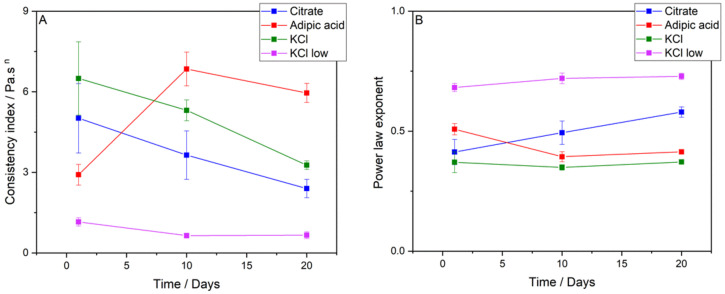
The change in power law parameters consistency index, *k* (Panel (**A**)), power law exponent, and *n* (Panel (**B**)) as a function of time.

The change in viscosity of the 0.0075 M KCl system over the storage period was not large compared to the other emulsion systems, as highlighted by a decrease in the parameter *k* from 1.15 ± 0.15 Pa∙s^n^ to 0.66 ± 0.13 Pa∙s^n^ (shown in Figure 6A). The decrease in viscosity is most likely the result of oil droplet coalescence, as evidenced by the droplet size analysis in Section 3.2. An increase in droplet size of the dispersed phase serves to decrease the viscosity of the system [25,31]. The 0.1 M citrate buffer and 0.35 M KCl systems showed a decrease in viscosity over time. For the 0.1 M citrate buffer system, *k* decreased from 5.02 ± 1.29 Pa∙s^n^ to 2.40 ± 0.34 Pa∙s^n^, while for the 0.35 M KCl emulsions, *k* decreased from 6.50 ± 1.36 Pa∙s^n^ to 3.27 ± 0.16 Pa∙s^n^. The particle size data suggest no appreciable coalescence within the oil phase, meaning that these mechanisms are unlikely to be the underlying cause of the decrease in viscosity. However, the state of aggregation of these suspensions may well vary over time, and this will not always be revealed by particle size measurement of a diluted emulsion (such as the data presented in Figure 2). Based on the rheology data for both the 0.35 M KCl and 0.1 M citrate systems, the emulsions have a lower degree of aggregation as the emulsion ages.

The change in viscosity over time for the 0.1167 M adipic acid emulsions was different to that of the other emulsion systems. On day 1, the *k* parameter was 2.91 ± 0.39 Pa∙s^n^, increasing to 6.85 ± 0.63 Pa∙s^n^ on day 10, before decreasing slightly to 5.96 ± 0.35 Pa∙s^n^ on day 20. The overall increase in viscosity is likely to be the result of an increase in aggregation of the oil droplets in the emulsion over time, potentially due to some effect of 0.1167 M adipic acid on the emulsifiers in the system, or their interfacial population.

Figure 6B shows the change in the power law exponent, *n*, over time. The *n* values for the 0.35 M KCl emulsions remained unchanged over the storage period (*n* = 0.37 ± 0.04 on day 1 and *n* = 0.37 ± 0.01 on day 20). The 0.1167 M adipic acid emulsions experienced a decrease in *n* over time, from 0.51 ± 0.02 on day 1 to 0.41 ± 0.01 on day 20. For the 0.0075 M KCl emulsions, *n* increased slightly from 0.68 ± 0.02 on day 1 to 0.73 ± 0.01 on day 20. Likewise, the *n* value for 0.1 M citrate buffer increased with time from 0.41 ± 0.05 to 0.58 ± 0.02. An increase in *n* means that the sample is becoming slightly less shear-thinning [31].

### 3.5. Effect of Pectin Chemistry on Anthocyanin Colour and Leakage

The results from Section 3.1, Section 3.2, Section 3.3 and Section 3.4 indicate that the best performance of the electrolyte systems in terms of W_1_/O/W_2_ emulsion stability and anthocyanin colour retention was the 0.1 M citrate buffer system. As such, this electrolyte composition was chosen for further analyses, whereby the pectin chemistry (unmodified or amidated) was analysed for any effects on colour retention and anthocyanin encapsulation efficiency and leakage. Section 3.1 and Section 3.2 show a movement of W_1_ droplets to the W_2_ phase occurring due to W_1_ droplet expulsion and, as highlighted in Section 3.3, this is detrimental for colour stability. An analysis was performed to determine the colour retention of anthocyanins in the presence of both unmodified and amidated pectin, and whether the effect is a result of anthocyanin retention within the W_1_ phase. Anthocyanin retention within the W_1_ phase was determined via Equation (3); UV–Vis was used to quantify anthocyanins in the W_2_ phase, which was compared to the amount of anthocyanin added at the time of formulation. The process requires a 50:50 dilution of the W_1_/O/W_2_ emulsion with the background electrolyte.

**Figure 7:**
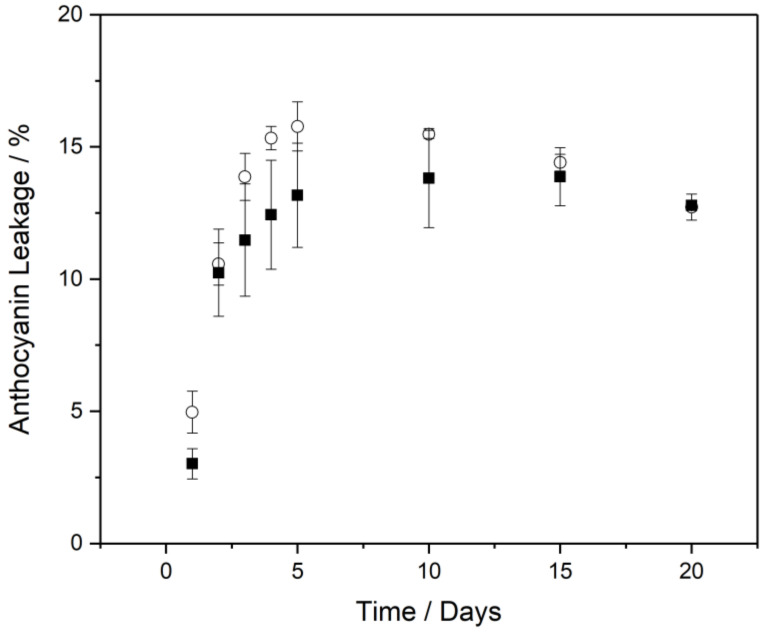
Anthocyanin leakage as a function of time, expressed as the amount (in %) of W_1_ anthocyanins located in the W_2_ phase. Leakage for unmodified pectin emulsions is represented by open circles; amidated pectin results are represented by closed squares.

There was a marginal difference in the encapsulation efficiency of anthocyanins due to the type of pectin used to gel the W_1_ phase. Unmodified pectin emulsions returned an encapsulation efficiency of 95.0 ± 0.8%, while the amidated pectin returned an encapsulation efficiency of 97.0 ± 0.6%. The results indicate that at the time of production, the amidated pectin allowed for greater retention of anthocyanins within the W_1_ droplets. This difference could be a result of more extensive gelation within the amidated pectin system or increased binding of anthocyanins to amidated pectin. Figure 7 shows the percentage of anthocyanins (added to the W_1_ phase at the time of formulation) present within the W_2_ phase as a function of time and pectin type. Initially, there is a difference between the two pectin types, with pectin amide (3.0 ± 0.6%) yielding a lower concentration of anthocyanins in the W_2_ phase than unmodified pectin (5.0 ± 0.8%). The greatest change in anthocyanin leakage from W_1_ to W_2_ occurred over the first 24 h of storage. This leakage of anthocyanins during the first 24 h aligns with our analysis of the CLSM images in Figure 1 and the droplet size distribution data in Figure 2A. During the first 24 h, we saw the greatest extent of W_1_ droplets being expelled into the W_2_ phase. Anthocyanin content within the W_2_ phase peaked on day 5 for unmodified pectin (15.8 ± 0.9%) and day 15 for amidated pectin (13.9 ± 1.1%), suggesting slower leakage of anthocyanins due to the presence of amidated pectin. However, as time elapsed, the difference in anthocyanin leakage between the two pectin types became less clear: when at day 20, the amount of anthocyanin in the W_2_ phase was identical for both systems (12.8 ± 0.1% for amidated pectin and 12.7 ± 0.5% for unmodified pectin). The average leakage values were lower for amidated pectin, however, the results were skewed by a single pectin amide emulsion having far greater encapsulation stability. Given the sample size of *n* = 3, there is no conclusive proof that amidated pectin is beneficial in terms of preventing anthocyanin leakage from the W_1_ to W_2_ phases.

**Figure 8:**
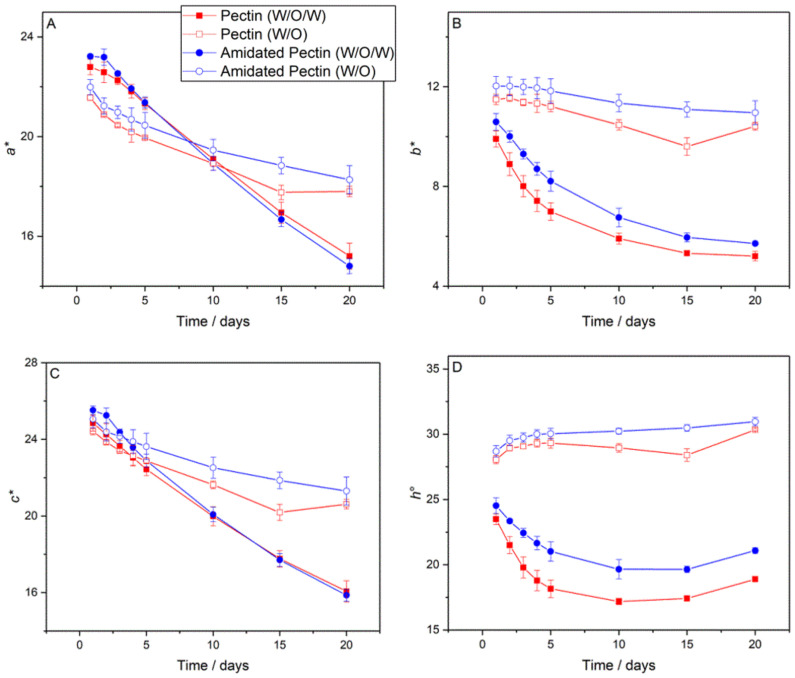
The effect of pectin chemistry on (**A**) a*; (**B**) b*; (**C**) c*; and (**D**) *h°*. Samples containing unmodified pectin are shown as squares (red), samples containing amidated pectin are shown as circles (blue), W_1_/O/W_2_ emulsions are represented with by closed symbols, and W_1_/O emulsions by open symbols.

The a*, b*, c*, and *h°* values as a function of storage time for the W_1_/O/W_2_ emulsions containing either unmodified or amidated pectin are presented in Figure 8. There was no significant difference in the a* value (Figure 8A) for the unmodified and amidated pectin emulsions at any point over the 20-day storage period. At the time of formulation, the a* value was 23.22 ± 0.09 for amidated pectin W_1_/O/W_2_ emulsions and 22.79 ± 0.31 for unmodified pectin emulsions. By day 20, the a* values decreased to 14.80 ± 0.31 for amidated pectin emulsions and 15.20 ± 0.52 for unmodified pectin emulsions. There was a small difference in the b* values for the amidated and unmodified pectin emulsions (Figure 8B), with the values being slightly lower for the unmodified pectin system. The b* values of the W_1_/O/W_2_ emulsions at the time of production were 10.59 ± 0.33 for amidated pectin emulsions and 9.91 ± 0.32 for unmodified pectin. By day 20, the b* values decreased to 5.71 ± 0.05 for amidated pectin emulsions and 5.20 ± 0.19 for unmodified pectin emulsions. The simultaneous decrease in a* and b* resulted in an overall decrease in c* (Figure 8C). Initially, the c* value for amidated pectin emulsions was 25.52 ± 0.22, marginally higher than that of unmodified pectin emulsions at 24.85 ± 0.41. By day 20, there was no significant difference in the c* values, meaning that the amidated pectin emulsions experienced a greater decrease in c* (a decrease from 25.5 on day 1 to 16 on day 20) than the unmodified pectin emulsions (a decrease from 24.5 at day 1 to 16 on day 20). The differences in a* and b* (Figure 8A,B) between the two pectin systems caused a subsequent difference in *h°* (Figure 8D), with the unmodified pectin system having lower values to that of the amidated pectin system. The *h°* values decreased (from initial values of 24.52 ± 0.60° for amidated pectin emulsions and 23.5 ± 0.40° for unmodified pectin emulsions) until around day 15 when the values began to increase. The increase in *h°* resulted from the change in b* being less pronounced compared with that of a* (Equation (2)). At day 20, the *h°* values were 21.08 ± 0.21° for amidated pectin emulsions and 18.89 ± 0.04° for unmodified pectin emulsions. In terms of W_1_/O/W_2_ emulsions, there was little difference in the a* and c* parameters for the unmodified and amidated pectin systems over the 20-day storage period. However, differences in the b* parameter also resulted in noticeable changes to *h°*.

The differences in colour parameters for the W_1_/O emulsions formulated with either amidated or unmodified pectin were less noticeable than that of the W_1_/O/W_2_ emulsions. The magnitude and change in the parameters as a function of storage time differed between the W_1_/O and W_1_/O/W_2_ emulsions, similar to the results in Section 3.4. In terms of a*, there was little difference between the two W_1_/O formulations with the amidated pectin emulsions experiencing a decrease in a* from 21.98 ± 0.30 to 18.27 ± 0.56 (a decrease of 16.90%), and the unmodified pectin emulsions experiencing a decrease from 21.56 ± 0.10 to 17.80 ± 0.20 (a decrease of 17.44%). Additionally, there was little difference in b*; the amidated pectin emulsions decreased from 12.03 ± 0.38 to 10.96 ± 0.48 (8.92% decrease) and the unmodified pectin emulsions decreased from 11.49 ± 0.20 to 10.42 ± 0.15 (9.31% decrease). The absence of any noticeable difference between the a* and b* parameters of the two emulsion formulations meant there was no significant difference between either the c* or *h°* values over the 20-day storage period.

Analysis of anthocyanin colour retention yielded relatively few differences between emulsions formulated with amidated pectin or unmodified pectin in the W_1_ phase. For W_1_/O/W_2_ emulsions, the main point of difference between the two pectin systems was the b* parameter, which was lower overall for unmodified pectin emulsions. This, in turn, affected the *h°* values and signified a minor difference in the colour produced by each emulsion. Taking into account the anthocyanin leakage from the W_1_ to W_2_ phases, as shown in Figure 7, there is not enough evidence to suggest that either amidated or unmodified pectin has any benefit in the stabilisation of anthocyanins within the formulated W_1_/O/W_2_ emulsion systems tested.

The differences in colour parameters for the W_1_/O emulsions (Figure 8) formulated with either amidated or unmodified pectin were less noticeable than that of the W_1_/O/W_2_ emulsions. The magnitude and change in the parameters as a function of storage time differed between the W_1_/O and W_1_/O/W_2_ emulsions, similar to the results in Section 3.4. In terms of a*, there was little difference between the two W_1_/O formulations, with the amidated pectin emulsions experiencing a decrease in a* from 21.98 ± 0.30 to 18.27 ± 0.56 (a decrease of 16.90%), and the unmodified pectin emulsions experiencing a decrease from 21.56 ± 0.10 to 17.80 ± 0.20 (a decrease of 17.44%). Additionally, there was little difference in b*; the amidated pectin emulsions decreased from 12.03 ± 0.38 to 10.96 ± 0.48 (8.92% decrease) and the unmodified pectin emulsions decreased from 11.49 ± 0.20 to 10.42 ± 0.15 (9.31% decrease). The absence of any noticeable difference between the a* and b* parameters of the two emulsion formulations means that there was no significant difference between either the c* or *h°* values over the 20-day storage period. In terms of W_1_/O emulsions, there was no difference in the colour retention of the two systems (i.e., there is no difference in the ability of the two pectin varieties to stabilise anthocyanins within W_1_/O emulsions).

## 4. Conclusions

The results of this study demonstrate the significant differences in anthocyanin and emulsion stability arising from the electrolyte phase composition in single and double emulsions. This was made apparent from the colour analysis of both water-in-oil (W_1_/O) emulsions and water-in-oil-in-water (W_1_/O/W_2_) emulsions, highlighting the need to understand the influence of aqueous phase composition on overall double emulsion stability (and precursor single emulsion stability) before determining the ability of complex emulsions to protect encapsulated molecules. Droplet sizing and rheology data revealed the differences in emulsion stability as a function of electrolyte composition; an electrolyte phase consisting of 0.1 M citrate buffer allowed for the best performance in terms of anthocyanin colour retention in W_1_/O and W_1_/O/W_2_ emulsions as well as stable droplet size and emulsion rheology. While the electrolyte composition effects were clear, there was no significant influence of pectin chemistry on the stability and protection of anthocyanins.

## Data Availability

Data is available from the corresponding author upon request.

## Supplementary Materials

The following are available online at https://www.mdpi.com/article/10.3390/foods11010034/s1, Table S1: A summary of the pH and conductivity values of the background electrolyte, W_1_ and W_2_ solutions for each composition. Conductivity is in mS.cm^−1^. Table S2: Quantitative analysis of major anthocyanins in MEDOX^®^ expressed as cyanidin-3-glucoside equivalents. Figure S1: Volume-weighted diameter percentiles d(0.1), d(0.5), and d(0.9) for: (A) 0.1 M citrate buffer; (B) adipic acid; (C) 0.35 M KCl; and (D) 0.0075 M KCl. d(0.1) is represented by circles, d(0.5) by squares, and d(0.9) by triangles. Average values calculated from three independent measurements (including error bars) are shown. Figure S2: A schematic of the CIE*L*a*b** colour system, highlighting the parameters relevant to this work, namely: *a**, *b**, *c** and *h°*, Figure S3: The CIE*L*a*b** colour parameters *a** (Panel A) and *b** (Panel B) for W_1_/O emulsions. The 0.1 M citrate buffer system is represented by circles, 0.1167 M adipic acid by squares, 0.35 M KCl by triangles, and 0.0075 M KCl by diamonds. Figure S4: Chroma (*c*,* Panel A) and hue angle (*h°*, Panel B) for W_1_/O emulsions calculated from the data in Figure S3. The 0.1 M citrate buffer system is represented by circles, 0.1167 M adipic acid by squares, 0.35 M KCl by triangles, and 0.0075 M KCl by diamonds. Figure S5: Flow curves between shear rates of 1 and 100 s^−1^ for 0.1167 M adipic acid (red squares) and 0.35 M KCl (purple diamonds) emulsions on days 1 (closed symbols) and 20 (open symbols). Figure S6: Digital photograph of the formed double emulsions, made using amidated pectin (left sample has a higher concentration of NaCMC, which was not used for the data presented in this work).
