# Supplementary material for: Influence of Aqueous Phase Composition on Double Emulsion Stability and Colour Retention of Encapsulated Anthocyanins
Source: Foods. 2021 Dec 23;11(1):34. doi: 10.3390/foods11010034 (PMC8750255; doi:10.3390/foods11010034)
Supplement: Supplementary file 1 [file foods-11-00034-s001.zip › foods-1499300-supplementary.pdf]

Solution Properties

The three electrolyte systems (0.1M citrate buffer, 0.1167M adipic acid, and 0.0075M KCl) had conductivity values of 5.8 mS.cm^{-1}, 1.3 mS.cm^{-1}, and 1.6 mS.cm^{-1}, respectively. The conductivity values along with the solution pH values are listed in Table S1. To test for ionic strength effects of simple 1:1 electrolyte on anthocyanin and emulsion stability, the data for 0.0075M KCl (1.6 mS.cm^{-1}) was compared with that of 0.35M KCl (43.5 mS.cm^{-1}).

Table S1. A summary of the pH and conductivity values of the background electrolyte, W_1 and W_2 solutions for each composition. Conductivity is in mS.cm^{-1}.

	**0.1M citrate buffer**		**0.1167M adipic acid**		**0.35M KCl**		**0.0075M KCl**	
	pH	Cond	pH	Cond	pH	Cond	pH	Cond
**Background**	3.5	5.8	3.6	1.3	3.5	43.5	3.5	1.6
**W_1**	3.6	7.8	3.6	3.6	3.5	42.5	3.8	3.9
**W_2**	3.8	7.6	4.0	3.7	5.1	39.1	5.5	4.0

Quantification of anthocyanins in MEDOX®

High performance liquid chromatography coupled with diode-array detection and electrospray ionization tandem mass spectrometry (HPLC-DAD-MS) was used to identify and quantify anthocyanins in MEDOX®. MEDOX® powder (1.0 mg) was dissolved in 1 ml 70% methanol containing 0.5% TFA. A 100µl aliquot of the MEDOX® sample solution was further diluted with 80 µl of the methanol/TFA solution and cyanidin (20 µl of a 100 ppm solution) was added as an internal standard for a final concentration of 10 ppm. The combined solution was vortexed for 30 s then centrifuge at 10,000 ×*g* for 10 min. The supernatant was transferred to HPLC vials for analysis. A series of standard solutions (2.5, 5.0, 10.0, 20.0, 40.0, and 80.0 ppm) were prepared via dilution of a 100 ppm cyanidin-3-O-glucoside stock solution in 70% methanol containing 0.5% TFA. Cyanidin was once again added as the internal standard at a concentration of 10 ppm. Analyses were performed on an HPLC-DAD-QQQ LC/MS instrument (G6495c triple quadrupole mass spectrometer, Agilent technologies, Santa Clara, USA). Chromatographic separation was performed using an Agilent Poroshell 120 SB-C18 column (100 mm × 2.1 mm × 2.7 µm) (Agilent Technologies, Santa Clara, USA), and the temperature of the column oven was maintained at 30 °C. The eluents were water (formic acid 5%, v/v) (A) and acetonitrile (B), with a gradient of 5–18% B (0–10 min), 18–30% B (10–15 min), 30–90% B (15–18 min), 90% B (18–19 min), 90–5% (19–21 min), then the column was re-equilibrated for 3 min at 5% B, at a flow rate of 0.3 $ml.min^{-1}$. The DAD detection was set at 525 nm. The experimental conditions of the QQQ detection were as follows: ESI interface; positive mode; nebulizer, 35 psi; gas temperature, 220 °C; gas flow rate, 18 $L.min^{-1}$; sheath gas flow rate, 12.0 $L.min^{-1}$; sheath gas temperature, 300 °C; capillary voltage, 3000 V; mass range from m/z 100 to 1000. All data were processed using the Masshunter

Qualitative and Quantitative analysis software version 10.0 (Agilent Technologies, Santa Clara, USA).

MEDOX® anthocyanin supplement is produced from the extract of bilberries and black currants. As shown in Table S2, the total anthocyanin content in the MEDOX® powder is 8.76 ± 0.33 % (w/w, Cyanidin-3-glucoside equivalents /dry mass). Moreover, twelve different anthocyanins were identified. Cyanidin glycoside (-rutinoside,-hexoside and -pentoside) and delphinidin glycoside are the most abundant anthocyanins, comprising 77.8% of total anthocyanin content.

Table S2**.** Quantitative analysis of major anthcoyanins in MEDOX® expressed as cyanidin-3-glucoside equivalents.

	Average (% w/w, Cyanidin-3-glucoside equivalents /dry mass)	SD
Delphinidin-hexoside 1	0.63	0.02
Delphinidin- hexoside 2	1.36	0.05
Cyanindin- hexoside 1/Dephinidin- rutinoside	2.77	0.10
Cyanindin- hexoside 2	0.79	0.03
Petunidin- hexoside 1	0.14	0.01
Cyanindin-rutinoside / Cyanindin -pentose	1.90	0.07
Petunidin- hexoside 2	0.34	0.01
Petunidin-pentose	0.22	0.02
Malvidin- hexoside 1	0.27	0.01
Malvidin- hexoside 2	0.34	0.01
Sum	8.76	0.33

Droplet size analysis

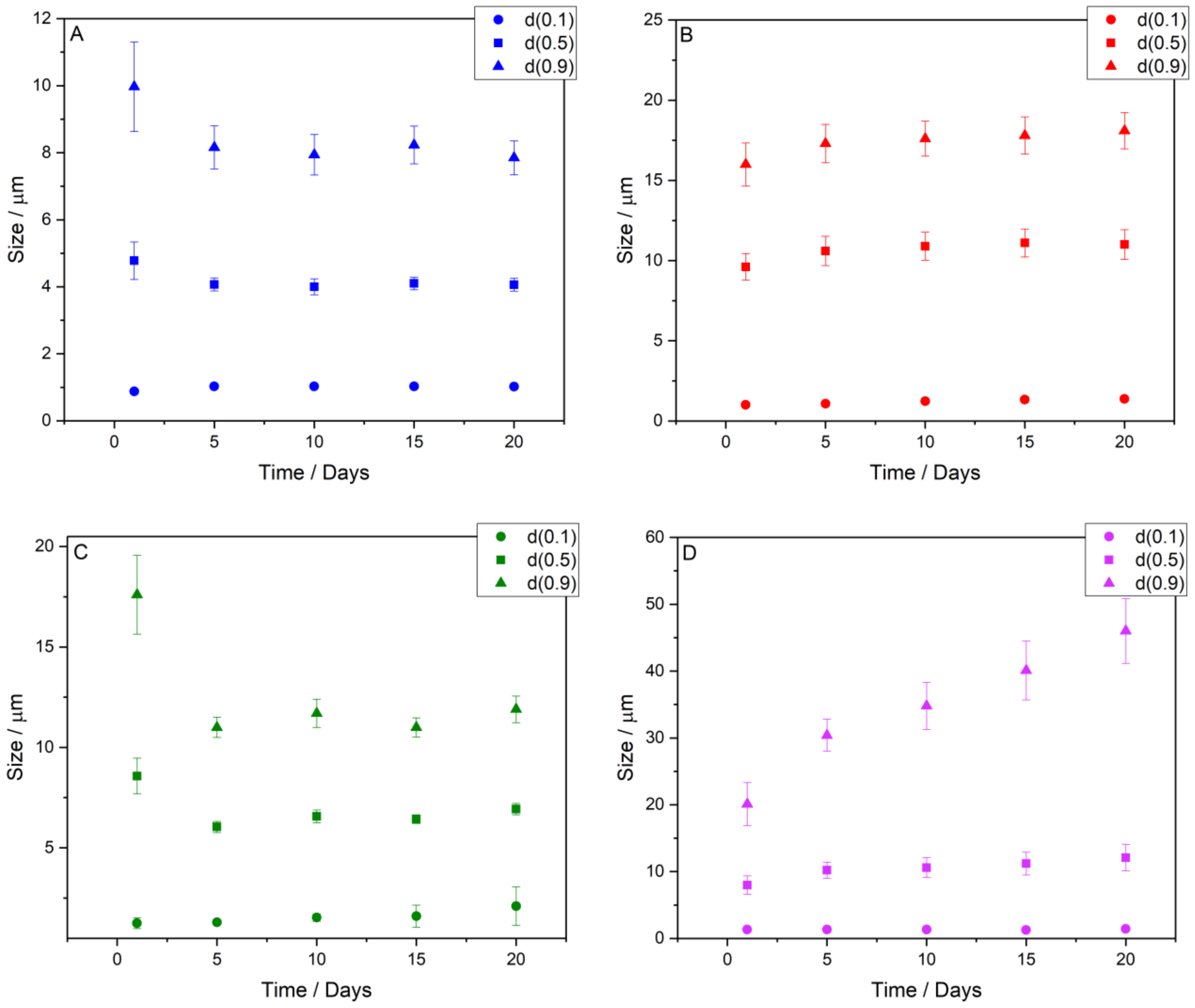

Figure S1. Volume-weighted diameter percentiles d(0.1), d(0.5), and d(0.9) for: (A) 0.1M citrate buffer; (B) adipic acid; (C) 0.35M KCl; and (D) 0.0075M KCl. d(0.1) is represented by circles, d(0.5) by squares, and d(0.9) by triangles. Average values calculated from three independent measurements (including error bars) are shown.

Colour analysis

Chroma (*c**) is a measure of colour vibrance, with higher values indicating higher colour intensity, while the hue angle (*h*°) is attributed to perceived colour [1] i.e. where a colour sits with respect to the CIE*L*a*b** colour wheel (Figure S2). A hue angle of 0° indicates red and a value of 90° indicates yellow [2].

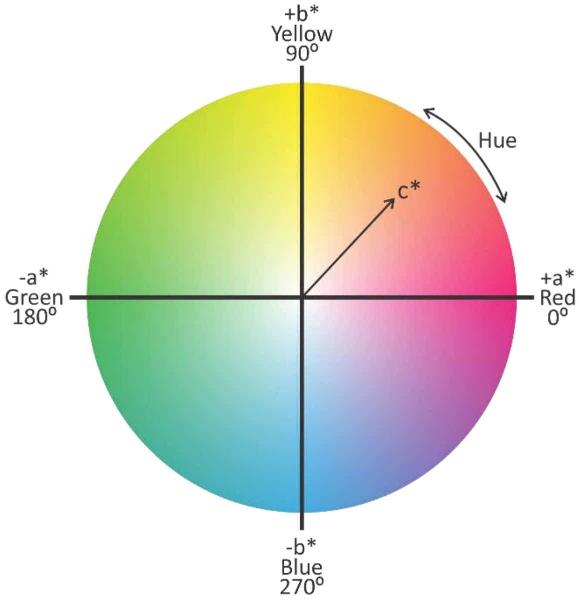

Figure S2. A schematic of the CIE*L*a*b** colour system, highlighting the parameters relevant to this work, namely: *a**, *b**, *c** and *h*°.

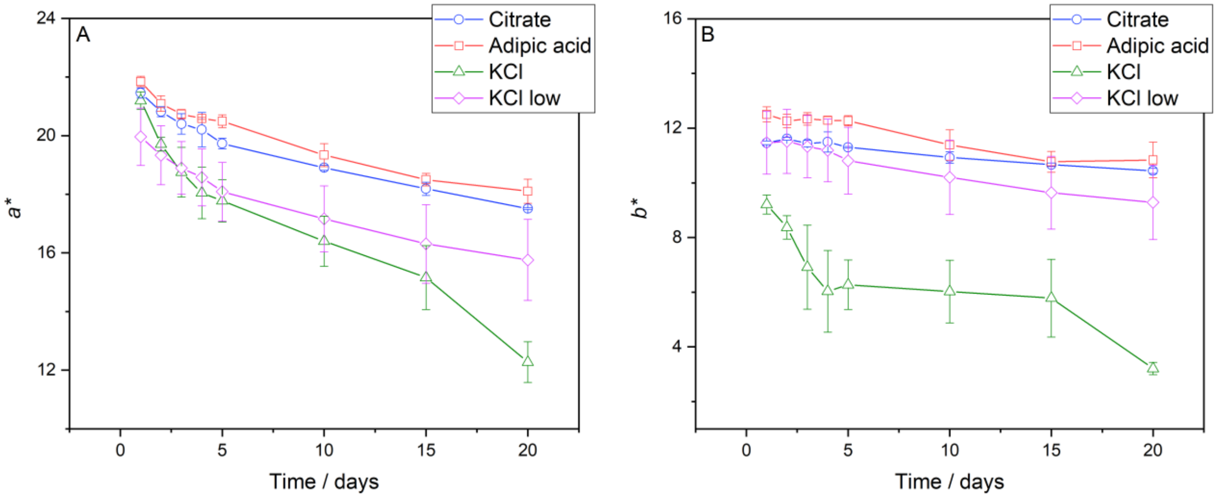

Figure S3. The CIE*L*a*b** colour parameters *a** (Panel A) and *b** (Panel B) for W_1/O emulsions. The 0.1M citrate buffer system is represented by circles, 0.1167M adipic acid by squares, 0.35 M KCl by triangles, and 0.0075M KCl by diamonds.

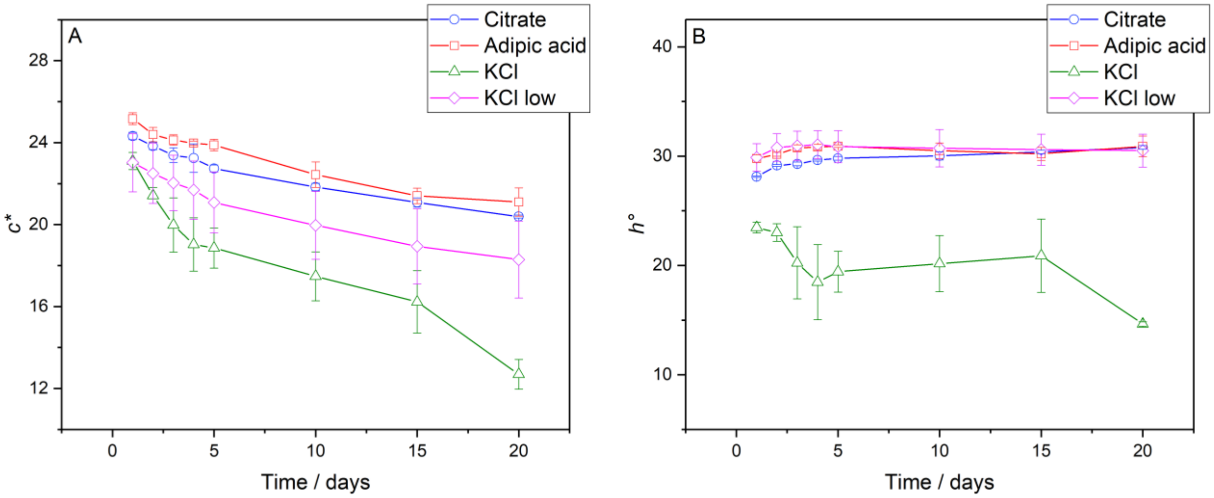

Figure S4. Chroma (*c**, Panel A) and hue angle (*h*°, Panel B) for W_1/O emulsions calculated from the data in Figure S3. The 0.1M citrate buffer system is represented by circles, 0.1167M adipic acid by squares, 0.35 M KCl by triangles, and 0.0075M KCl by diamonds.

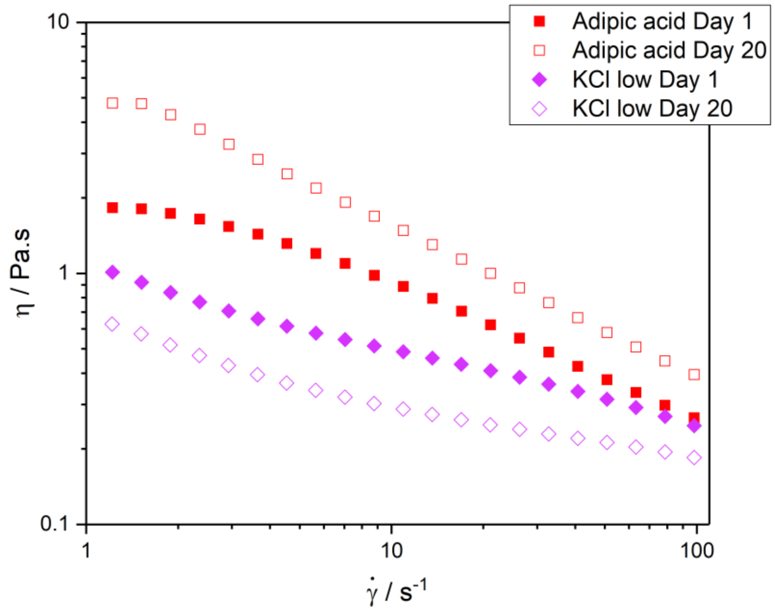

Figure S5. Flow curves between shear rates of 1 and 100 s^{-1} for 0.1167M adipic acid (red squares) and 0.35M KCl (purple diamonds) emulsions on days 1 (closed symbols) and 20 (open symbols).

Visual Appearance of Double Emulsions

An example photograph of a formed double emulsion is provided in Figure S6. There are two samples presented, with the variation being the concentration of NaCMC used in the formulation (2 wt% or 1.5 wt%, with the latter concentration being the one used for the work presented in this manuscript). The emulsions were physically stable (i.e. no phase separation) throughout the entirety of the 20 day investigation period, with only an alteration in perceived colour being detected visually. No image is provided of the emulsion at 20 days, as the colour analysis provided in the manuscript is the pertinent (and quantitative) data for that colour alteration.

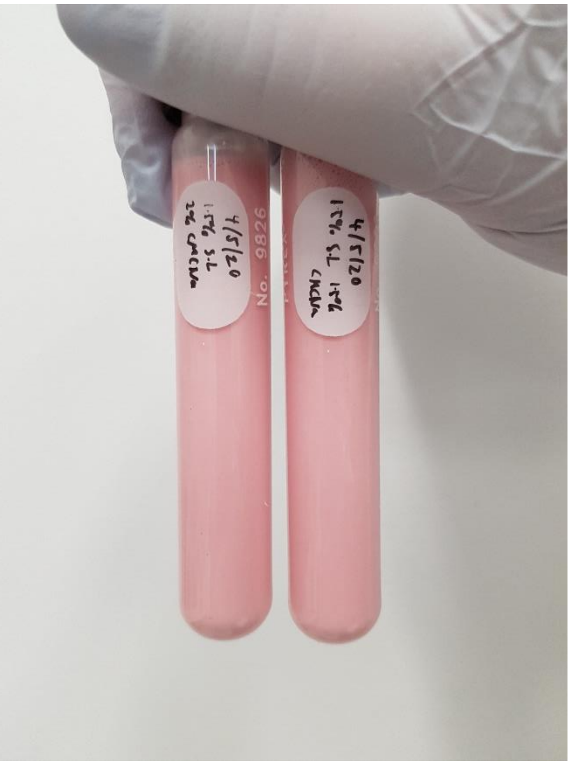

Figure S6. Digital photograph of the formed double emulsions, made using amidated pectin (left sample has a higher concentration of NaCMC, which was not used for the data presented in this work).

References

1. Shaddel, R.; Hesari, J.; Azadmard-Damirchi, S.; Hamishehkar, H.; Fathi-Achachlouei, B.; Huang, Q. Use of gelatin and gum Arabic for encapsulation of black raspberry anthocyanins by complex coacervation. *International Journal of Biological Macromolecules* **2018**, *107*, 1800-1810, doi:https://doi.org/10.1016/j.ijbiomac.2017.10.044.
2. de Almeida Paula, D.; Mota Ramos, A.; Basílio de Oliveira, E.; Maurício Furtado Martins, E.; Augusto Ribeiro de Barros, F.; Cristina Teixeira Ribeiro Vidigal, M.; de Almeida Costa, N.; Tatagiba da Rocha, C. Increased thermal stability of anthocyanins at pH 4.0 by guar gum in aqueous dispersions and in double emulsions W/O/W. *International Journal of Biological Macromolecules* **2018**, *117*, 665-672, doi:https://doi.org/10.1016/j.ijbiomac.2018.05.219.
